# An Approach to Improve Specimen Processing for the Flexural Strength Testing of Zirconia

**DOI:** 10.3390/ma17143479

**Published:** 2024-07-14

**Authors:** Nashib Pandey, Sabrina Karlin, Michael Marc Bornstein, Nadja Rohr

**Affiliations:** 1 Biomaterials and Technology, Department Research, University Center for Dental Medicine Basel UZB, University of Basel, 4058 Basel, Switzerland; 2 Department of Periodontics & Oral Implantology, College of Medical Sciences, Bharatpur 44207, Nepal; 3 Department of Oral Health & Medicine, University Center for Dental Medicine Basel UZB, University of Basel, 4058 Basel, Switzerland

**Keywords:** zirconia, 3Y-TZP, surface treatment, biaxial flexural strength, three-point bending test

## Abstract

Measuring the flexural strength of restorative materials such as zirconia is crucial for providing proper indications for clinical applications and predicting performance. Great variations in specimen preparation for flexural strength measurements exist among laboratories. The aim was to evaluate how the processing method, surface treatment, and test method of the specimens affect the flexural strength of zirconia. Zirconia specimens (VITA YZ HT) (*n* = 270) were processed using CAD/CAM or were conventionally milled with three different surface treatments (machined, ground, polished) and were measured with three-point bending (non-chamfered/chamfered) or biaxial flexural strength test. Weibull statistics were conducted. The mean flexural strength values ranged from 612 MPa (conventional, machined, three-point bending non-chamfered) to 1143 MPa (CAD/CAM, polished, biaxial flexural strength). The highest reliability is achieved when specimens are prepared using thoroughly controllable processing with CAD/CAM and subsequently polished. Higher strength values are achieved with the biaxial flexural strength test method because the stress concentration in relation to the effective volume is smaller. Polishing reduces surface microcracks and therefore increases the strength values.

## 1. Introduction

Zirconia (ZrO_2_) is widely used in restorative dentistry as a framework material or monolithically for single crowns and fixed dental prostheses [1,2,3,4]. Zirconia is a biocompatible oxide ceramic revealing a high fracture toughness and flexural strength [5,6]. The polycrystalline material can appear in three crystal phases as follows: monoclinic, tetragonal, and cubic, depending on various factors such as the temperature, pressure, and dopants added [7,8]. At ambient temperatures, pure zirconia stabilizes in the monoclinic phase, while the tetragonal and cubic phases become stable with the addition of dopants with lower valences than zirconium (Zr) to create oxygen anion vacancies [7,8]. The zirconia used in dentistry as a framework material is mainly stabilized in the tetragonal phase by adding 3 mol% of yttria (Y_2_O_3_) and is known as 3Y-TZP (3 mol% yttria stabilized tetragonal zirconia polycrystal) [9].

Zirconia exhibits high strength owing to a unique crack-prevention mechanism called transformation toughening [10,11]. This means that, when stress is applied to 3Y-TZP, the tetragonal crystal phase transforms to monoclinic near the crack tip, forming a transition zone. This phase transformation from tetragonal to monoclinic is accompanied by a volume increase of 3–5%, preventing crack propagation [10,12].

To predict the clinical performance of a restorative material and to suggest the proper indication, mechanical properties such as flexural strength or fracture toughness are measured. However, to determine the flexural strength of zirconia, a wide range of varying methods are available, including the three-point bending test, four-point bending test, and biaxial flexural strength test. To conduct these tests, specimens with specific dimensions are to be prepared. For the three-point bending test, rectangular bars are required, while disc-shaped specimens are needed to measure the biaxial flexural strength [13,14,15]. Although there is an ISO 6872 available describing the specimen dimensions, the available blank dimensions of the zirconia material may not fit the proposed dimensions [16,17]. Additionally, varying laboratory equipment used for processing, polishing, and sintering and various specimen holders used for testing may impair the outcome [18,19,20,21,22,23,24,25,26].

It is not known if the strength is affected by the cutting method from the blank by either computer-aided design/computer-aided manufacturing (CAD/CAM) or conventional milling. Additionally, the strength is highly sensitive to the applied polishing procedure as polishing removes microcracks within the superficial layer and consequently increases strength [18,19,27]. To polish the specimen surface according to ISO 6872, it is suggested to use a diamond grinding wheel (grain size: 30 μm to 40 μm) and a diamond suspension (grain size: 15 μm to 20 μm) [18]. However, the testing of machined specimens is also possible if the shape and dimensions meet specifications. Additionally, chamfering along the long axis of the three-point bending specimens to minimize grinding damage or chipping is recommended [16]. A previous flexural strength test with a 3-TZP material has shown that even though polishing steps are followed according to a pre-defined protocol, variances in flexural strength were severe among the 12 included laboratories [15].

The specimen preparation of 3Y-TZP for flexural strength measurements seems to be highly sensitive to various factors, and guidance is needed on how specimens should be prepared to achieve the highest strength values. The aim of this study was to evaluate how the cutting method, surface treatment, and strength test affect the flexural strength values of 3Y-TZP. The flexural strength of 3Y-TZP was obtained on specimens prepared for three-point bending and biaxial flexural strength tests that were either produced with CAD/CAM or conventionally milled with three different surface treatments (machined, ground, polished). As an additional outcome, it was tested if chamfering the edges of three-point bending test specimens increases the strength. The research hypotheses were that (1) there is no difference between cutting methods in flexural strength, (2) the flexural strength increases with additional polishing steps, and (3) there is no difference in the flexural strength between three-point bending non-chamfered, chamfered, and biaxial flexural strength tests.

## 2. Materials and Methods

**Figure 1:**
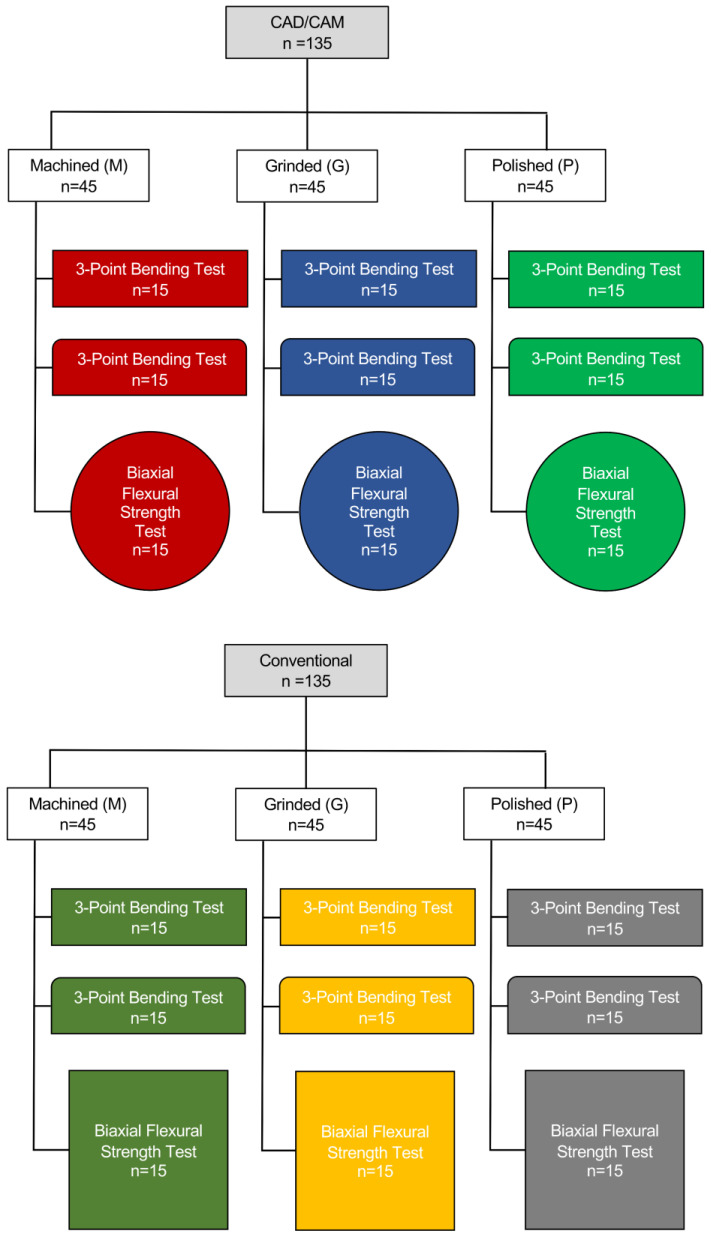
Test set-up for 3Y-TZP specimen preparation based on the specimen processing method (CAD/CAM vs. conventional), polishing procedure applied (machined, ground, polished), and flexural strength testing method (3-point bending test, biaxial flexural strength). The shape of the flexural strength test boxes represents specimen dimensions of the 3-point bending test of non-chamfered bars and chamfered bars and for the biaxial flexural strength test of circular and rectangular discs.

The flexural strength of 3Y-TZP (VITA YZ HT; VITA; Bad Säckingen, Germany) was determined by using either a three-point bending strength test set-up or a biaxial flexural strength test with three different surface treatments (machined, ground, polished). For the 3-point bending test, it was tested if chamfering the edges of the bar-shaped specimens influences the flexural strength. Specimens for the CAD/CAM group were prepared using circular discs (ø 98.4 mm × 14 mm), while conventional specimens were prepared using rectangular blocks (40/19). The zirconia discs (LOT Nr. 83290) and blocks (LOT Nr. 99290) were from the same batch. The test set-up of the different groups of specimens as per the specific cutting method, test method, and surface treatment applied is displayed below (Figure 1).

### 2.1. Specimen Preparation

#### 2.1.1. Three-Point Bending Test

For the specimen preparation for the 3-point bending test, the bar-shaped dimensions of the final dimensions after the sintering and surface treatments of 4.0 mm × 20.0 mm × 1.2 mm (±0.1 mm) were prepared by either CAD/CAM or conventional milling with a wire saw. For the CAD/CAM group, bars were virtually designed overdimensionally (Meshmixer V 3.5; Autodesk Inc., San Francisco, CA, USA). The stereolithography (STL) file was nested for CAM (Programill PM7; Ivoclar AG and Programill CAM 2021 V4.4.13.0; Ivoclar Digital; Schaan, Liechtenstein) and milled with 4 rods at the end of each bar. 

For the conventional processing group, specimens were milled with a diamond wire saw machine (wire 0.28 mm; Well Walter Ebner; Le Locle, Switzerland) under constant water irrigation to obtain square cross-sections of 20 mm × 19 mm × 15 mm that were consequently sectioned to the specimen dimension. The blocks were therefore fixed on the holder using sculpting wax (Esprit; al dente; Goslar; Germany). To test the effect of chamfering the edges, all groups were produced in duplicates and, before sintering, the chamfering of the edges of 0.1 mm of the treated surface was conducted with silicon carbide paper grit 1200 (Struers; Ballerup, Denmark) for half of the specimens (*n* = 15 per group).

#### 2.1.2. Biaxial Flexural Strength Test

For the group CAD/CAM, disc-shaped specimens with a final diameter of 12.5 mm and a height of 1.2 mm after sintering and surface treatment were produced. STL files were nested and milled with 3 supporting rods per specimen as described above. For the group conventional processing, milling round disc-shaped specimens out of rectangular blocks with the diamond saw was not possible; therefore, it was decided to use rectangular dimensions of 12.0 mm × 12.0 mm × 1.2 mm as previously described [19,20,21]. Blocks were mounted on the holders of the diamond wire saw (Well Walter Ebner) and cut as above for the 3-point bending test specimens.

### 2.2. Surface Treatments

All prepared specimens were divided into the following groups for further surface treatments:

- Machined (M): No surface treatment was applied after cutting the specimens.
- Ground (G): Manual grinding was carried out with silicon carbide paper grit 1200 without water-cooling before sintering.
- Polished (P): Manual grinding was performed with silicon carbide paper grit 1200 (without water-cooling), followed by grit 2500 and grit 4000 with water-cooling using a polishing machine (Minitech 363; Presi SA; Eybens, France). After sintering, additional polishing was conducted using diamond paste (6 µm; Presi SA) on a silicon cloth (DP-Nap; Struers) mounted on a polishing machine (Labopol 5; Struers).

### 2.3. Sintering

Before sintering, the specimens were stored in a drying oven (Biostar Ministar; Scheu Dental; Iserlohn, Germany) for 30 min. Sintering was conducted (VITA Zyrcomat 6100 MS program VITA YZ HT Universal; VITA Zahnfabrik) with the following parameters: the sintering oven was heated at a rate of 17 °C for 47 min until 1000 °C. It was then again heated at a rate of 17 °C for 26 min to reach 1450 °C, and then the furnace was left at this temperature for 2 h. Then, it was cooled down to 1200 °C, to 900 °C, and finally to 200 °C with a total time taken of 3 h 13 min.

### 2.4. Flexural Strength Testing 

The 3-point bending and biaxial flexural strength testing was carried out according to ISO 6872 [18]. Each specimen dimension was recorded using a Digital Caliper, (TESA-Cal IP 67; TESA, Renes, Switzerland, accuracy: ±0.03 mm). The parallelism of the specimens were checked visually, and non-parallel specimens were prepared again. The holding device for 3-point bending tests consisted of support rollers (1.5 mm in diameter), arranged with a span of 12.0 mm. The force was applied midway between the two support rollers via a third roller (1.5 mm in diameter). The test specimens were placed with the pre-treated surface facing the two support rollers in the device and loaded until fracture with a cross-head speed of 1 mm/min with a universal testing machine (Z020; Zwick/Roell, Ulm, Germany). The flexural strength σ was calculated in MPa using the following equation:

σ = 3Fl/2wb^2^.

where

F is the breaking force in N.

l is the test span (center distance between the support rollers) in mm (=12.0 mm),

w is the width of the test specimen in mm,

b is the thickness of the test specimen.

The specimen holder for the biaxial flexural strength test comprised three tempered steel balls with a diameter of 3.2 mm. The steel balls formed an equilateral triangle with an edge length of 10 mm, and the ball support circle was 120°. The specimens were placed with the surface-treated side facing towards the steel balls in the holder. After the positioning, the specimens’ center was loaded from above with a plunger of tempered steel with a diameter of 1.4 mm until failure with a cross-head speed of 1 mm/min in a universal testing machine (Z020; Zwick/Roell).

The flexural strength was calculated according to the following equation:

σ = −0.2387 F(X − Y)/d^2^.

where

σ is the biaxial flexural strength,

F is the breaking force in N,

coefficients X and Y with

X = (1 + υ) ln[(r_2_/r_3_)]^2^ + [(1 − υ)/2] (r_2_/r_3_)^2^.

Y = (1 + υ) [1 + ln(r_1_/r_3_)^2^] + (1 − υ) (r_1_/r_3_)^2^.

where

υ is the Poisson’s ratio (=0.25),

r_1_ is the support (mean) contact diameter (mm) (=10.0 mm),

r_2_ is the (mean) loaded contact diameter (mm) (=1.4 mm),

r_3_ is the diameter of the specimen (mm) (=12.5 mm),

d is the thickness.

### 2.5. Surface Characterization

#### 2.5.1. Surface Roughness

Surface roughness parameters were obtained for each specimen with a 3D laser scanning microscope (VK-X1000 Series; Keyence, Tokyo, Japan). The surface roughness parameters Sa (arithmetical mean height) and Sz (maximum height) were analyzed using ×20 objective and laser light (intensity) over an area of 2500 µm × 1500 µm.

#### 2.5.2. Scanning Electron Microscopy

Scanning electron microscopy (SEM) (XL-30 ESEM; Philips, Eindhoven, The Netherlands) was used to visualize the microstructure and topography of the specimens from each group. Before SEM, specimens were sputtered with gold (SCD 005 Sputter Coating Unit; BalTec, Pfäffikon, Switzerland). Imaging was performed at an acceleration of 20 kV and magnifications of ×500, ×2000, ×5000, and ×10,000.

### 2.6. Statistical Analysis

The specimen size was chosen according to ISO 6872 [18]. To describe the reliability of the specimens, Weibull two-parameter distribution was applied to all flexural strength data according to ISO 6872 [18]. The Weibull modulus and Weibull characteristic strength were calculated. Mean and standard deviations were calculated for the flexural strength measurements, as well as the surface roughness values (Sa, Sz). Strength data were tested for normal distribution using the Shapiro–Wilk test. Three-way ANOVA followed by a Bonferroni post hoc test was applied to analyze the effects of the specimen processing method, surface treatment, and flexural strength test method on the flexural strength values (α = 0.05) (StatPlus Pro V6 (2016); AnalystSoft; Walnut, CA, USA).

## 3. Results

### 3.1. Flexural Strength

The overall flexural strength values for specimens processed by CAD/CAM (929 ± 172 MPa) were significantly higher than for the conventional specimens (871 ± 209 MPa) (*p* < 0.001). However, within the sub-groups, only values within the three-point bending test non-chamfered group were significantly higher (*p* < 0.001), and no difference was found within the three-point bending test chamfered (*p* = 0.570) and the biaxial flexural strength test (*p* = 0.238) groups. The surface treatment also affected the flexural strength significantly (*p* < 0.001), with the highest values for polished (1012 ± 166 MPa) followed by ground (910 ± 189 MPa) and machined surfaces (778 ± 148 MPa). Overall, significantly higher flexural strength values were obtained when using the biaxial flexural strength test (1016 ± 167 MPa) than when using the three-point bending strength test with chamfered specimens (884 ± 151 MPa) and non-chamfered specimens (800 ± 196 MPa) (all *p* < 0.001) (Table 1 and Table 2).

**Figure 2:**
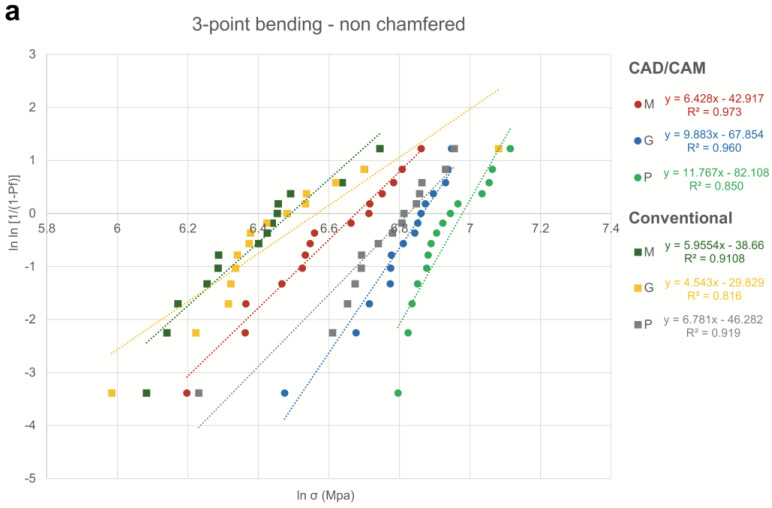

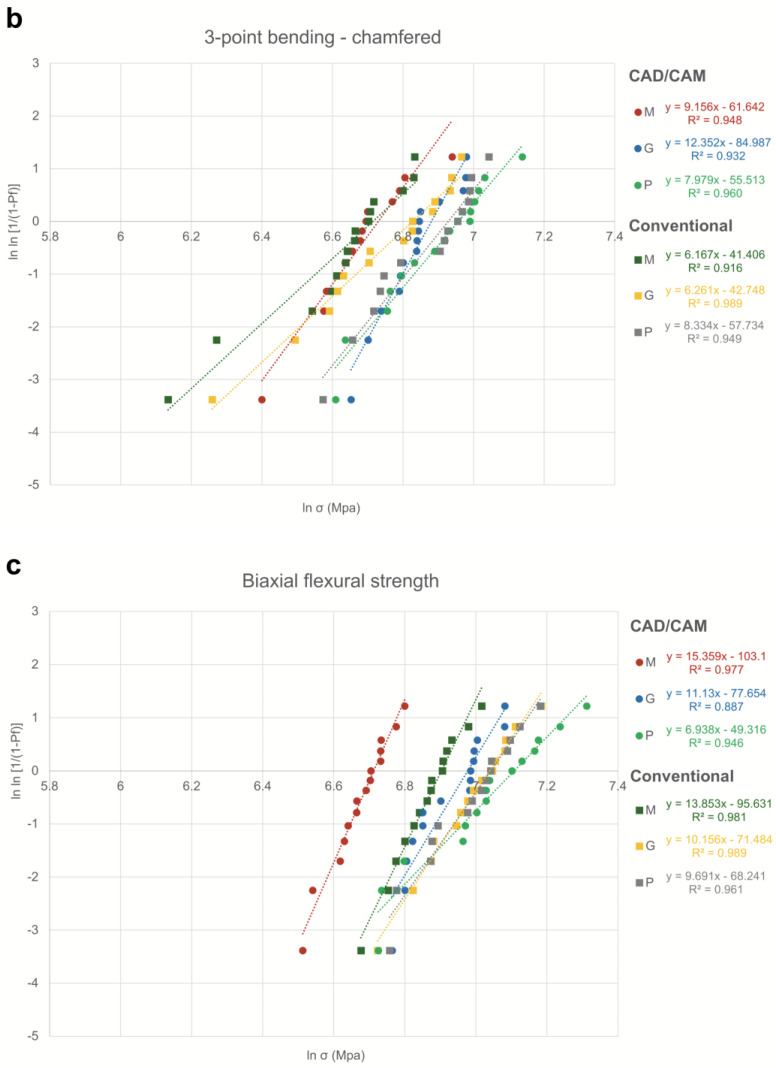
Weibull plots of flexural strength values for zirconia. Specimens were processed either with CAD/CAM or conventional processing and surfaces were subsequently ground (G), machined (M), or polished (P) before applying (**a**) 3-point bending tests using non-chamfered specimens, (**b**) 3-point bending tests using chamfered specimens, or (**c**) biaxial flexural strength tests using circular discs for CAD/CAM and rectangular discs for conventional.

High Weibull modulus values are an indicator of high reliability and, therefore, even the distribution of defects within the specimen volume. The highest values were observed for CAD/CAM specimens that were polished for all three-point bending test non-chamfered (Weibull modulus 6.98) and chamfered (Weibull modulus 6.96) groups, as well as biaxial flexural strength (Weibull modulus 7.10) groups. For specimens of the three-point bending test that were not chamfered, additional surface treatment severely improved Weibull modulus values, especially for conventionally processed specimens. Weibull modulus values were highest overall when conducting the biaxial flexural strength test, followed by the three-point bending test with chamfered specimens and the three-point bending test with non-chamfered specimens (Figure 2).

### 3.2. Surface Characterization

The linear correlation between the mean values of surface roughness Sa and mean flexural strength values was weak (R^2^ = 0.164). Overall, the Sa and Sz decreased with additional polishing steps (Table 3).

**Figure 3:**
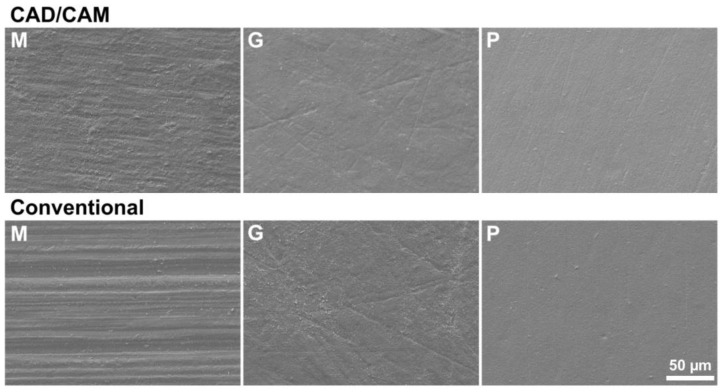
Scanning electron microscope images (×500) of surfaces of the specimens after 3-point bending testing. Upper row exhibit CAD/CAM processed specimens, lower row conventional, M: Machined, G: Ground, P: Polished. Additional polishing steps result in smoother surface topographies.

**Figure 4:**
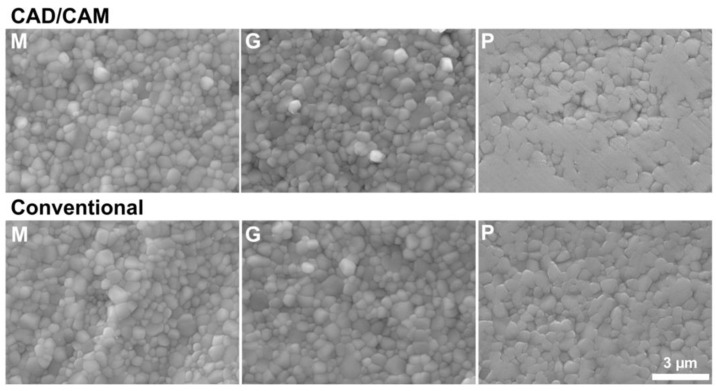
Scanning electron microscope images (×10,000) of surfaces of specimens after 3-point bending testing. Upper row CAD/CAM, lower row Conventional, M: Machined, G: Ground, P: Polished. Grain growth is visible on all surfaces, while grain boundaries are fused due to the polishing procedure on P.

The surface topography of specimens for three-point bending and biaxial flexural strength tests displayed a similar surface topography; therefore, only SEM images for three-point bending are displayed at ×500 (Figure 3) and ×10,000 (Figure 4). Machined surfaces revealed anisotropic topographies with aligned grooves. On ground surfaces, grooves appeared less deep and were not oriented. Polished surfaces seemed smooth with hardly visible grooves in SEM images captured at ×500 (Figure 3). Crystal growth is visible on all surfaces, occurring when a threshold temperature is exceeded during sintering (Figure 4). The boundaries of the individual grains were fused on polished surfaces, especially on CAD/CAM-processed specimens’ topographies.

## 4. Discussion

Measuring the flexural strength of restorative dental materials such as zirconia is crucial for providing the proper indication for clinical use and predicting their respective performance. As a great variety exists among laboratories regarding the specimen preparation protocols, the present in vitro study evaluated how the cutting method, surface treatment, and applied strength testing affect flexural strength values of 3Y-TZP. A higher flexural strength overall was achieved with specimens prepared using CAD/CAM; however, differences within the test methods were only significant for non-chamfered specimens of the three-point bending test. Therefore, the first hypothesis of this study was partially rejected. The second hypothesis, stating that the flexural strength increases with additional polishing steps, was confirmed for all groups. The flexural strength obtained with the test method biaxial flexural strength showed significantly higher values, followed by the three-point bending test using chamfered specimens, resulting in a rejection of the third hypothesis.

According to the ISO 6872 for ceramic materials in dentistry, zirconia is a type II ceramic and is further divided into a class between 1 and 5, based on the flexural strength and chemical solubility that determine the clinical indication [18]. For fixed dental prosthesis (FDPs) up to three units in the anterior and premolar region, a flexural strength of 300 MPa is required (class 3), while for FDPs in the molar region, 500 MPa is needed (class 4). FDPs with more than three units necessitate a strength higher than 800 MPa (class 5). The chemical solubility is to be <100 µg/cm^2^. However, ISO 6872 allows the manufacturer to select any standard specimen geometry for the flexural strength testing of ceramics. If the tested material achieves a satisfactory mean strength value with any of the described flexural strength test methods, then the product passes the requirements. This study revealed that the mean flexural strength values can vary from 612 to 1143 MPa for the same 3Y-TZP depending on specimen processing, surface treatment, and the applied testing methods. Zirconia is a brittle material that is highly sensitive to flaws in processing and needs to be handled with great care for restorations in order to not impair its mechanical strength [27]. The manufacturer approved the 3Y-TZP tested in the present study as a class 5 material with a strength of 1200 MPa (three-point bending test chamfered), as depicted in the respective scientific information sheet, and reported a Weibull modulus of 14. The obtained mean flexural strength value for polished and chamfered three-point bending test specimens in this study was 990 MPa with a Weibull modulus of 7. Previous studies reported flexural strength values of 1155 ± 88 (CAD/CAM, machined, three-point bending test, non-chamfered) [22], 1106 ± 97 MPa (conventional, polished, three-point bending test, chamfered plus heat-treatment) [23], and 1170 ± 63 MPa (CAD/CAM, machined, biaxial flexural strength) [24] for the same 3Y-TZP. Values within this range (1143 ± 192 MPa) in the present study were measured for the CAD/CAM group, where the surface of the specimens were polished, and biaxial flexural strength testing performed. This was also the group achieving the highest strength overall. 

In the present study, it was highlighted that the specimen processing, surface treatment, as well as the applied strength test method clearly affect the measurements. The interaction between specimen processing and surface treatment as well as the strength test method was significant (Table 1), meaning that specimen processing seems to be the most important factor affecting the flexural strength values. Hence, as each laboratory is conducting the strength measurements with slightly different approaches, and due to the high sensitivity of zirconia to defects in the pre-sintered state, varying strength values are to be expected [15,16,17,23,24].

Overall, preparation with CAD/CAM has shown to provide more reliable results than conventional milling as specimen grinding is automated. Flaws may occur during the fixation of the specimens on the milling holders, and the effect of water-cooling is still unclear. However, the available zirconia blank dimensions and milling machines may disable CAD/CAM specimen processing according to the recommended dimensions [19]. To obtain the flexural strength of zirconia available in discs, the most reliable and convenient method for providing the highest flexural strength values is the processing of disc-shaped specimens using CAD/CAM, polishing the surfaces, and testing these with biaxial flexural strength tests. For zirconia materials that are only available as blocks, conventional milling into square discs that are used for biaxial flexural strength testing as previously suggested [19,20] is an alternative option, providing higher flexural strength values than when three-point bending tests are applied for these specimens. When the three-point bending test is chosen, producing specimens with CAD/CAM is encouraged, followed by polishing and chamfering to minimize edge defects and consequently increase flexural strength values. The result that higher flexural strength values were achieved with biaxial flexural strength testing compared to the three-point bending testing of specimens was confirmed in previous studies with 3Y-TZP [16,25]. Bar-shaped specimens that are used for the three-point bending strength test often have edge flaws, acting as stress concentration sites that lead to failure. For the biaxial flexural strength testing of specimens, edge flaws are not relevant as fractures originate from the material’s intrinsic flaws or polishing scratches [27]. Additionally, biaxial flexural strength specimens have a smaller effective volume, meaning that the volume under stress during loading is smaller than for the three-point bending test specimens.

Previously, a round-robin test with the biaxial flexural strength testing of zirconia at 12 different laboratories was conducted [15]. The specimens were CAD/CAM-ground at the same location out of a zirconia disc, and only the sintering and polishing steps were conducted at different laboratories. Large variations in flexural strength may have occurred due to the varying sintering parameters of the available furnaces at the other laboratories for machined specimens. The flexural strength was increased overall when specimens were polished, as observed in the present study. The monoclinic phase content that is induced by grinding during CAD/CAM is partially reverted by polishing [9,11,26]. Hence, the presence of the tetragonal phase is consequently increased, and the effect of phase transformation toughening may increase the strength of 3Y-TZP. In SEM images (Figure 4), it is also visible that the polishing procedure partially fused the crystal grain boundaries, eliminating potential crack initiation points. In the round-robin test with polished specimens, variations in flexural strength among laboratories were even larger than for machined specimens. This was probably due to the additional processing step that was performed slightly differently at the laboratories, with an apparent effect on flexural strength [15]. Specimen handling, especially in the sensitive pre-sintered state of zirconia, force application during polishing, and varying polishing protocols among laboratories are difficult to standardize due to the available equipment. After conducting this study, an automated polishing machine was acquired (Tegramin; Struers), and CAD/CAM-processed specimens of the same material were polished in fixable holders for a standardized pressure application using diamond suspensions of 9 µm, 3 µm, and 1 µm after sintering. Using this automated polishing protocol, the surface roughness was lowered to Sa of 0.09 µm, and the flexural strength values and reliability increased for biaxial flexural strength specimens (1244 ±133 MPa, Weibull modulus 7.2), but remained similar for three-point bending test specimens that were chamfered (1030 ± 134 MPa, Weibull modulus 7.0).

Although the surface roughness decreased with additional polishing steps, no linear correlation was found between flexural strength values and surface roughness parameters Sa and Sz. This finding was confirmed in a previous study for biaxial flexural strength values of conventionally milled specimens that were ground, polished, or glazed, as well as within the round-robin test [15,26]. It needs to be emphasized that surface roughness parameters Sa and Sz provide only limited information on the actual surface topography of the specimens that potentially influences flexural strength. Additionally, 3Y-TZP is an unpredictable material with its phase transformation capability that is affected by numerous additional factors, such as the particle size of the pre-sintered material, heat treatment, or surface treatments that all have an impact on the materials’ surface and mechanical behavior.

## 5. Conclusions

Based on the results of this in vitro study, the following conclusions were drawn:

1. Specimens processed by CAD/CAM rather than conventional milling result in higher flexural strength values, especially with non-chamfered bar-shaped specimens measured using the three-point bending test.
2. Polishing procedures are to be applied to increase the flexural strength of specimens.
3. Biaxial flexural strength testing results in significantly higher flexural strength values than a three-point bending testing method. For three-point bending tests, the chamfering of specimens is recommended to reduce edge defects and consequently increase flexural strength values.

## Figures and Tables

**Table 1:** Flexural strength mean values (MPa) and standard deviations for specimens that were either CAD/CAM or conventionally processed. Specimen surfaces were either machined, ground, or polished. Groups fulfilling the strength requirements of ISO 6872 for class 5 ceramics (>800 MPa, indication FDPs > 3 units) are marked dark grey and light grey for class 4 ceramics (>500 MPa, indication FDPs < 3 units).

		CAD/CAM			Conventional		
		Machined	Ground	Polished	Machined	Ground	Polished
**3-point bending test**	non-chamfered	740 ± 135 ^A,a^	912 ± 104 ^B,a^	1027 ± 102 ^C,a,b^	612 ± 123 ^D,a^	648 ± 181 ^A,D,a^	859 ± 133 ^B,a^
	chamfered	794 ± 104 ^A,a^	934 ± 90 ^B,C,a^	990 ± 146 ^C,a^	765 ± 129 ^A,b^	858 ± 154 ^A,B,b^	963 ± 133 ^B,C,a^
**Biaxial flexural strength test**		796 ± 62 ^A,a^	1024 ± 107 ^B,b^	1143 ± 192 ^C,b^	960 ± 84 ^B,c^	1086 ± 128 ^B,C,c^	1087 ± 133 ^B,C,b^

Statistical differences within subgroups are indicated with differing superscript letters (uppercase horizontal comparison, lowercase vertical comparison) (*p* < 0.05).

**Table 2:** Results of three-way ANOVA test of flexural strength values.

Factor	Df *	SS ^#^	MS ^$^	F	*p*-Value
**Specimen processing**	1	226,898	226,898	13.78	<0.001
**Surface treatment**	2	2,479,665	1,239,832	75.31	<0.001
**Strength test**	2	2,141,199	1,070,600	65.03	<0.001
**Specimen processing × Surface treatment**	2	124,237	62,118	3.77	0.024
**Specimen processing × Strength test**	2	675,136	337,568	20.50	<0.001
**Surface treatment × Strength test**	4	116,040	29,010	1.76	0.137
**Specimen processing × Surface treatment × Strength test**	4	142,597	35,649	2.17	0.073
**Within Groups**	252	4,148,928	16,464		
**Total**	269	10,054,699	37,378		

* degree of freedom, ^#^ sum of squares, ^$^ MS mean square.

**Table 3:** Mean values and standard deviations of surface roughness parameters Sa (arithmetical mean height) and Sz (maximum height of profile) in µm.

	CAD/CAM			Conventional		
	Machined	Ground	Polished	Machined	Ground	Polished
**3-point bending test non-chamfered**						
**Sa**	1.48 ± 0.38	0.46 ± 0.04	0.38 ± 0.07	0.84 ± 0.14	0.53 ± 0.11	0.43 ± 0.12
**Sz**	17.74 ± 3.76	9.70 ± 6.53	6.05 ± 2.65	13.28 ± 4.17	13.93 ± 6.10	9.23 ± 5.28
**3-point bending test chamfered**						
**Sa**	1.82 ± 0.41	0.53 ± 0.02	0.39 ± 0.05	0.93 ± 1.23	0.56 ± 0.19	0.48 ± 0.08
**Sz**	22.91 ± 4.39	16.76 ± 12.13	8.07 ± 8.37	15.31 ± 7.41	7.19 ± 1.7411	11.83 ± 13.38
**Biaxial flexural strength test**						
**Sa**	1.15 ± 0.20	0.62 ± 0.14	0.20 ± 0.05	2.06 ± 0.81	0.55 ± 0.06	0.24 ± 0.03
**Sz**	31.85 ± 11.29	32.89 ± 12.77	4.34 ± 1.28	22.20 ± 14.78	13.60 ± 11.59	8.96 ± 6.11

Sa arithmetical mean height, Sz maximum height of profile.

## Data Availability

The original contributions presented in the study are included in the article, further inquiries can be directed to the corresponding author.
